# Tourism guide cloud service quality: What actually delights customers?

**DOI:** 10.1186/s40064-016-3345-4

**Published:** 2016-10-04

**Authors:** Shu-Ping Lin, Chen-Lung Yang, Han-Chung Pi, Thao-Minh Ho

**Affiliations:** 1 Department of Technology Management, Chung Hua University, Hsinchu, Taiwan; 2 Ph. D. Program of Technology Management, Chung Hua University, Hsinchu, Taiwan

**Keywords:** Tourism guide cloud, Overall satisfaction, Loyalty, Service quality, Enjoyment

## Abstract

**Background:**

The emergence of advanced IT and cloud services has beneficially supported the information-intensive tourism industry, simultaneously caused extreme competitions in attracting customers through building efficient service platforms. On response, numerous nations have implemented cloud platforms to provide value-added sightseeing information and personal intelligent service experiences. Despite these efforts, customers’ actual perspectives have yet been sufficiently understood. To bridge the gap, this study attempts to investigate what aspects of tourism cloud services actually delight customers’ satisfaction and loyalty.

**Methods:**

336 valid survey questionnaire answers were analyzed using structural equation modeling method.

**Results:**

The results prove positive impacts of function quality, enjoyment, multiple visual aids, and information quality on customers’ satisfaction as well as of enjoyment and satisfaction on use loyalty.

**Conclusions:**

The findings hope to provide helpful references of customer use behaviors for enhancing cloud service quality in order to achieve better organizational competitiveness.

## Introduction

In recent years, tourism has become one of the most profitable and dynamic pillar industries worldwide (Zhou et al. 2014), generating an estimated 11 % of the global gross domestic products and is currently employing 200 million people and serving 700 million tourists globally, a figure which is expected to double by the year 2020 (Amato et al. 2014). Noteworthy, the emergence of advanced IT and cloud technology has beneficially transferred the traditional tourism into currently information-synchronized tourism industry with high extent of freedom, flexibility, and productivity (Zhou et al. 2014). For these advantages, cloud services have been perceived a better solution for meeting customer expectations and enhancing satisfaction (Bilgihan et al. 2011; Chen and Tseng 2013).

On response to tourists’ increasing demands for information, various cloud technology-integrated applications have been developed in different nations, for instance the “Smarter Tourism” and “Smart Tourism Destination” in China (Zhang et al. 2012), the Context Cloud infrastructure in Spain (Martin et al. 2012), the touristic context-aware recommendation system in Italy (Amato et al. 2014). Especially, Tourism Bureau and Institute for Information Industry in Taiwan have implemented the “Smart Tourism Taiwan service platform” and “Map of Taipei Amusement” (MOTA) to provide tourists with sightseeing information and personal intelligent service experiences. These endeavours have in turn created abundant opportunities for promoting cloud services in tourism industry. Nonetheless, despite great efforts in implementing cloud platforms, customers’ actual perspectives toward value-added cloud services have yet been sufficiently understood.

Previous studies on examining cloud services from end-consumer perspectives have commonly applied and extended IT adoption theories such as TAM model (Davis et al. 1989; Venkate sh and Davis 2000) and the IS success model (DeLone and McLean 2004) to explore the relationships among various constructs such as trust, satisfaction, and service convenience (Chen et al. 2015), cost, opportunities and risks (Martens and Teuteberg 2012), cloud service quality (Benlian et al. 2011), and end-users’ motivation, privacy concerns, entrepreneurial orientation, ethics and cloud computing adoption (Ratten 2012). However, the extant literature has stressed that loyalty and other potential factors are important constructs to consider for IT adoption-related studies (Cyr et al. 2006; Flavian et al. 2006).

Nowadays, through cloud services, tourists request information irrespective of time and space by the use of mobile phones or portable devices; additionally, the information has to be personalized according to their real needs (Martin et al. 2012). For this reason, information quality has been suggested to considerably affect users’ satisfaction and continuous use toward a system (Chang and Chen 2008; Chen et al. 2015; DeLone and McLean 2004). Considering function quality, the storage capacity and system functions have been indicated to potentially lead to user dissatisfaction if not fulfilled (Lee et al. 2014). Moreover, since 91 % of unhappy customers may not purchase again and if the system provides timely feedback and appropriate solutions to complaints, approximately 82–95 % of customers may remain with a system (Faed et al. 2014), real feedback has been expected to influence user satisfaction. Furthermore, since multiple visual aids have also been argued to significantly increase dissatisfaction if not well-managed, customers’ sensory experience toward interface visual design has been proposed to be critical to their continuous use (Lorenzo-Romero et al. 2013; Tuch et al. 2010). On elaborating, in recent IT-related studies, a hedonic component namely ‘‘fun’’, ‘‘playfulness’’, or ‘‘enjoyment’’ has been incorporated to the TAM model in order to better explore user pleasure toward utilizing a system (Chung and Tan 2004; Cyr et al. 2006; Moon and Kim 2001). Finally, based on the DeLone and McLean IS success model, many studies have advocated that satisfaction can boost customer loyalty (Bayraktar et al. 2012; Lee et al. 2014; Siddiqi 2011). It has been additionally suggested that resolving the dissatisfaction will restore customers’ faith and loyalty (Daft and Lengel 1986). Nonetheless, despite potential relationships among these dimensions, few researches have emphatically investigated these relationships in the field of tourism cloud application. To bridge this gap, this study attempts to take Taiwan tourism industry to propose a tourism cloud-satisfaction and loyalty model by integrating the TAM model and the refined IS success model to investigate which factors of cloud services (i.e. information quality, function quality, real feedback, multiple visual aids, and enjoyment) actually delight customer satisfaction and loyalty. With the achieved findings, this study hopes to provide future research and industrial managers with helpful references of customer use behaviours and useful guidelines for enhancing cloud service quality in order to achieve better development and competitiveness.

 With the integration of tourism industry and cloud services, this study expects to provide considerable academic and practical contributions to the extant literature. Firstly, in spite of the fact that TAM and Delone and McLean IS models have been widely applied to explore customer satisfaction and use loyalty, focuses are mainly on ordinary IT platforms and in popular industry sectors such as e-commerce (Chen et al. 2015; Kim and Kankanhalli 2009), e-banking (Siddiqi 2011), mobile phone sector (Bayraktar et al. 2012), hospitality (Bilgihan et al. 2011), electronics usage (Chang and Chen 2008), and logistics and transport (Faed et al. 2014). A missing in in-depth investigations on the trendy cloud computing technology and tourism industry has been noticed. Second, despite recent attempts in examining cloud services in tourism industry, a brief review has revealed that cloud computing technology has been mainly paid attention to possible applications in establishing innovative systems such as the context-aware recommendation service system for supporting tourists’ path planning (Amato et al. 2014), the tourism guide context-aware mobile services (Martin et al. 2012), or e-tourism low-cost airline systems for backpacking (Zhou et al. 2014). Again, research missing is acknowledged concerning knowledge of cloud services’ attributes and their consequent impacts on users’ adoption and use loyalty since an IT platform cannot be designed and operated well without sufficient understanding of its characteristics. Therefore, this study with the holistic view on cloud service quality attributes from reliable perspectives of tourists has beneficially provided sufficient understanding of what factor actually delights customers, which in turn brings about significance in contributions in comparison with the extant literature.

 The remainders of this study continue with a review of tourism cloud development and dimensions of cloud services that are expected to have impacts on tourists’ overall satisfaction and loyalty in second section. Third sections presents data collection, construct measures, and research methodology, followed by statistical results in fourth section. In-depth managerial insights, discussions, and conclusions are stated in fifth section. This study ends with research limitations and future recommendations in sixth section.

## Literature review and Hypothesis development

### Tourism cloud development

In recent years, by overcoming a front-loaded investment in IT-related equipment and life-cycle maintenance investments, cloud computing has been considered a promising approach with significant cost advantages (Mohaupt and Hilbert 2013; Park and Ryoo 2013), seamless and secure access irrespective of time, space, and portable devices (Lin et al. 2009; Marston et al. 2011). With advanced functions and designs, cloud services can enable more proactive customer-to-business relationship as well as convenient network access with rapid information provision (Chen and Tseng 2013; Kalloniatis et al. 2014). Due to plentiful benefits, cloud technology has been widely applied to various service fields, especially in tourism industry as they efficiently meet customer expectations and promote their satisfaction (Bilgihan et al. 2011; Gopalani and Shick 2011) and open up new avenues for tourism service providers to deliver value-added services to tourists (Lee et al. 2014).

It has been observed that tourists nowadays begin their trip before they arrive, which brings about great competition in attracting customers through building web services or platforms that are sufficient, efficient, and available irrespective of places and time (Martin et al. 2012). On response, a wide range of countries have integrated cloud technologies into tourism activities in order to develop effective information platforms for tourists, for instance the “Smarter Tourism” and “Smart Tourism Destination” in China for timely providing location-based services and real-time, multi-directional experience among service providers and tourists (Zhang et al. 2012), the Context Cloud infrastructure in Spain with a web front-end where tourists’ context-aware mobile services can be easily configured using Google Maps layer and Drools Expert System (Martin et al. 2012), the touristic cloud context-aware recommendation system in Italy which is based on previous users’ experience, tourists’ personal preferences, and situational contexts for recommending touristic paths (Amato et al. 2014), etc.

### Theoretical development

#### Information quality and overall satisfaction

In the extant literature, information quality has been referred to as the IT user interface’s generation of relevant and accurate information in accordance with customer needs (Petter et al. 2013). As such, information quality encompasses the aspects of accuracy, precision, and timeliness (Chen et al. 2015).

In prior TAM and IS success-related studies, customer satisfaction has been defined as consumers’ positive post-purchase evaluation and responses to product or service experience (Lin and Wang 2006), being derived from the fact that the systems provide products or services that exceed users’ expectations (Raval 2010). Hence, customer satisfaction implies the systems’ effective endeavours in enhancing their value-added gains, which in turn stimulates their repurchase intention, word-of-mouth, and loyalty (Chou 2015).

Concerning the relationship between these two dimensions, previous studies have suggested that information quality and customer satisfaction are consistently correlated (Chen et al. 2015; DeLone and McLean 2004; Petter et al. 2013). Based on these premises, the first hypothesis was proposed as follows:

##### H1

Cloud services’ information quality has a positive impact on customer satisfaction.

### Function quality and overall satisfaction

Regarding function quality, cloud system’s storage capacity and the system functions has been indicated to potentially lead to user dissatisfaction if not fulfilled (Lee et al. 2014). Moreover, previous success IS studies have put forward that function quality influences users’ overall satisfaction through providing satisfactory storage capacity (Burda and Teuteberg 2014) as well as effective usability, availability, reliability, and adaptability (Chen et al. 2015; DeLone and McLean 2004). Hence, the following hypothesis was assumed:

#### H2

Cloud services’ function quality has a positive impact on customer satisfaction.

### Real feedback and users’ overall satisfaction

This study is based on prior studies to refer cloud system’s real feedback as timely responses and assistance to both customers’ complaints and requests (Chou 2015). Since approximately 91 % of unsatisfied customers may not conduct repurchase while about 82–95 % of them may remain with a system once being provided with timely feedback and assistance as well as being appropriately addressed regarding their complaints (Faed et al. 2014), real feedback has been expected to exert certain impact on user (DeLone and McLean 2004). In line with this, real-time travel assistance and feedback have been suggested to beneficially offer tourists with latest information relevant to their needs and motivate tourists—tourists’ conversations, which in turn dramatically affect their satisfaction (Lee et al. 2014). Accordingly, the following hypothesis was stated:

#### H3

Cloud services’ real feedback has a positive impact on customer satisfaction.

### Multiple visual aids and overall satisfaction

Previous studies have referred an IT system’s or user interface’s multiple visual aids to its emotional appeal and are expressed through colours mix and layout, shapes, font type and size, music, animation, photos, sound effects, text clarity, readability, etc. (Cyr et al. 2006). Noteworthy, the role of multiple visual aids on customer satisfaction remains controversial in the extant literature. Specifically, since multiple visual aids are used to deliver information, they are argued to significantly increase dissatisfaction if not well-managed (Daft and Lengel 1986; Lorenzo-Romero et al. 2013; Tuch et al. 2010); hence, customers’ sensory experience toward interface visual design has been proposed to considerably influence whether a user stays and shops (Cyr et al. 2006; Rosen and Purinton 2004). On contrast, since it has been argued that visual attributes are must-have basic qualities of web portals or IT systems (Lee et al. 2014), they will not affect user satisfaction. Therefore, in order to explore multiple visual aids’ actual impact on customer satisfaction, this study establishes the following hypothesis:

#### H4

Cloud services’ multiple visual aids have a positive impact on customer satisfaction.

### Enjoyment and customers’ overall satisfaction

The concept of ‘‘enjoyment’’ (i.e. “fun”, “playfulness”) has been recently incorporated to the TAM model in IT-related studies in order to better explore user pleasure toward utilizing a system (Chung and Tan 2004; Daft and Lengel 1986). Noteworthy, it has been put forward that customers’ enjoyment toward the use of a system positively affects their overall satisfaction and loyalty by clarifying that when individuals are in the enjoyment state, they will perceive the interaction intrinsically interesting as well as pleasure and playfulness, which in turn stimulates their positive attitude and repurchase intention toward the system (Cyr et al. 2006; Moon and Kim 2001). Therefore, two following hypotheses were suggested:

#### H5

Customers’ enjoyment toward cloud services has a positive impact on satisfaction.

#### H6

Customers’ enjoyment toward cloud services has a positive impact on their loyalty.

### Customers’ overall satisfaction and loyalty

Previous researches have widely defined customer loyalty to be favourable attitude toward a specific website or IT system and repetitive buying behaviour without intention to switch to another, which can be reflected through customers’ re-purchase behaviours, and purchase frequency (Chou 2015; Cyr et al. 2006; Flavian et al. 2006; Lin and Wang 2006).

Customer satisfaction of IT system has been indicated to positively affect customer loyalty (DeLone and McLean 2004; Gudigantala et al. 2011; Kalloniatis et al. 2014; Kim and Kankanhalli 2009; Shin et al. 2013; Song et al. 2012). Moreover, Tseng (2015) indicated that the popularization of Internet and the development of cloud computing have not only changed our lifestyles, but have impacted the ways in which enterprises relate with their customers and found web-based self-service (WBSS) satisfaction has a significant positive influence on WBSS continued usage intention. In other words, satisfied customers will be more likely to use the same IT system (including cloud computing system) of user interface again. Based on these studies, the following hypothesis was assumed:

#### H7

Customers’ satisfaction toward cloud services has a positive impact on their loyalty.

## Research methods

### Data collection

The development of Tourism Guide Cloud Service is still at the initial stage in Taiwan; thus, the users who understand this new technology and adopt it are limited. Therefore, individuals from the public over 16 years old with experience in using Tourism Guide Cloud Service were selected as the sample in this study. Through the convenience sampling method, after removing invalid ones (e.g. incomplete questionnaires), 336 valid questionnaires were collected, a return rate of 93.33 %. The profile of the respondents is shown in Table 1. Female consisted of 50.3 %, slightly higher than males 49.7 % of the respondents. Most of the respondents (73.2 %) are single in the marital status. Most of the respondents (57.4 %) fell into the 21–30 years age group. Most of the respondents had college education (68.5 %), and most of the respondents using Tourism Guide Cloud Service less than 1 year (59.8 %).

**Table 1:** Profile of the respondents (n = 336)

Characteristics	Item	Frequency	Percent (%)
Gender	Male	167	49.7
	Female	169	50.3
Age	Below 20 years old	39	11.6
	21–30 years old	193	57.4
	31–40 years old	55	16.4
	More than 41 years old	49	14.6
Marital status	Single	246	73.2
	Married	90	26.8
Education level	High school	20	5.9
	College	230	68.5
	Master	86	25.6
Using tourism guide cloud	Less than 1 year	201	59.8
	1–2 years	89	26.5
	2–3 years	39	11.6
	More than 3 years	7	2.1

### Measures of the constructs

Based on the Information Systems Success Model (DeLone and McLean 2004) and Media Richness Theory (Daft and Lengel 1986), this study selected 7 constructs as the basis to develop the integrated model for Tourism Guide Cloud Service. These constructs were Information quality (IQ), Function Quality (FQ), Real Feedback (RF), Multiple Visual Aids (MV), Enjoyment (E), Overall Satisfaction (OS), and Loyalty (L). Their definitions and measures (see Table 2) are as follows. All measures were assessed using the Likert 5-point scale (1 = “very dissatisfied”, 5 = “very satisfied”).

**Table 2:** Measures of the constructs

Variables/	Items
Information quality (IQ)	IQ1: timeliness
	IQ2: detailed
	IQ3: easy to understand
	IQ4: accuracy
	IQ5: completeness
	IQ6: professional
Function quality (FQ)	FQ1: wireless online
	FQ2: GPS
	FQ3: traffic information
	FQ4: instant update
	FQ5: navigation
	FQ6: itinerary planning
Real feedback (RF)	RF1: get others to share
	RF2: Share feelings
	RF3: quick response negative comments
Multiple visual aids (MV)	MV1: link with social networking
	MV2: link with video and audio
Enjoyment (E)	E1: pleasant
	E2: content intriguing
	E3: interesting content
Overall satisfaction (OS)	OS1: overall satisfaction
Loyalty (L)	L1: continued use
	L2: recommended use

### Data analysis methods

LISREL 8.12 was employed as the main instrument for data analysis. Confirmatory factor analysis (CFA) was utilized to examine reliability and validity of the measurement models, and then the structural model and proposed hypotheses were tested using structural equation modeling (SEM). Coefficient paths were estimated using the maximum likelihood method while the model’s overall fit was assessed using the following indicators of Chi square statistic/degrees of freedom (χ^2^/df), goodness-of-fit index (GFI), adjusted goodness-of-fit index (AGFI), normalized fit index (NFI), comparative fit index (CFI), root mean square error of approximation (RMSEA), and root mean square residual (RMR).

## Results

### Reliability and validity of the measurement model

In the measurement model, all multiple-item factors were firstly tested for reliability. The achieved Cronbach’s α coefficients ranged from 0.71 to 0.85 and construct reliability (CR) ranged from 0.71 to 0.87, which were all greater than the threshold 0.7 (Bagozzi and Yi 1988; Nunnally 1978), indicating high internal consistency and thus reliability for all indicators (see Table 3).

**Table 3:** Reliability and convergent validity analyses (n = 336)

Variables/Items	Mean	SD	Std factor loading	Cronbach’s α	CR
Information quality (IQ)	3.571			0.81	0.87
IQ1	3.573	0.738	0.72		
IQ2	3.531	0.741	0.81		
IQ3	3.564	0.743	0.72		
IQ4	3.573	0.738	0.70		
IQ5	3.540	0.776	0.72		
IQ6	3.534	0.843	0.73		
Function quality (FQ)	3.574			0.85	0.85
FQ1	3.494	0.795	0.66		
FQ2	3.536	0.842	0.67		
FQ3	3.664	0.809	0.70		
FQ4	3.571	0.789	0.76		
FQ5	3.708	0.779	0.67		
FQ6	3.473	0.850	0.69		
Real feedback (RF)	3.382			0.75	0.75
RF1	3.470	0.776	0.69		
RF2	3.420	0.749	0.69		
RF3	3.256	0.917	0.73		
Multiple visual aids (MV)	3.433			0.71	0.71
MV1	3.470	0.776	0.73		
MV2	3.40	0.762	0.76		
Enjoyment (E)	3.563			0.84	0.85
E1	3.586	0.798	0.76		
E2	3.548	0.748	0.83		
E3	3.557	0.774	0.83		
Overall satisfaction (OS)	3.691	0.673	1	–	–
Loyalty (L)	3.766			0.79	0.81
L1	3.729	0.661	0.93		
L2	3.803	0.684	0.70		

Confirmatory factor analysis (CFA) was then employed to examine construct validity of the measurement model. Furthermore, we used Mardia’s (1985) coefficient of multivariate kurtosis to assess multivariate normality, and the critical ratios of Mardia’s coefficient of multivariate kurtosis of all items are less than 1.96. Hence, the data belong to multivariate normal distribution. The model fit test results revealed all achieved values for fit indicators (χ^2^/df = 2.70, GFI = 0.83, AGFI = 0.80, NFI = 0.96, CFI = 0.98, RMSEA = 0.06, RMR = 0.05) meet the threshold recommended by Hu and Bentler (1998), indicating good model fit for the measurement model. Moreover, all achieved standardized factor loadings are greater than the threshold 0.50 (Bagozzi and Yi, 1988) (see Table 3). Overall, the convergent validity of all measurement indicators is validated (Hair et al. 2010).

A discriminant validity test is then performed to establish the distinction among the variables used in this study, and can be supported if the average variance extracted (AVE) is larger than the squared correlations between variables (Fornell and Larcker 1981). As shown in Table 4, all pairs of the squared correlations among latent constructs are smaller than the AVE of the respective variables. Hence, discriminant validity is supported.

**Table 4:** Discriminant validity analysis

Constructs	1	2	3	4	5	6	7
1. Information quality (IQ)	(0.54)						
2. Function quality (FQ)	0.23	(0.48)					
3. Real feedback (RF)	0.46	0.30	(0.50)				
4. Multiple visual aids (MV)	0.31	0.19	0.47	(0.56)			
5. Enjoyment (E)	0.44	0.26	0.42	0.44	(0.65)		
6. Overall satisfaction (OS)	0.25	0.12	0.29	0.23	0.44	(1)	
7. Loyalty (L)	0.20	0.12	0.20	0.24	0.23	0.58	(0.68)

The numbers in the lower triangular matrix are the squared correlations; the numbers in parentheses are AVE

### SEM results

This study aims to examine the factors influencing user’s overall satisfaction and loyalty in using Tourism Guide Cloud Service Quality. The SEM approach was used to interpret the measurement model. The overall fit indices of the proposed model were all greater than the thresholds recommended by Hu and Bentler (1998), indicating that the model had a good fit to the data (see Table 5). In addition, the R^2^ value of the unified model was 0.61 (>0.5), confirming high interpretability and good explanatory power.

**Table 5:** Fit indices for structural model

Fit indices	Recommended value	Structural model
GFI	≥0.9	0.83
AGFI	≥0.8	0.80
NFI	≥0.9	0.96
CFI	≥0.9	0.98
RMSEA	0 ≤ 0.1	0.06
RMR	≤0.05	0.046
*χ* ^*2*^ */df*	≤03	2.70

**Fig. 1:**
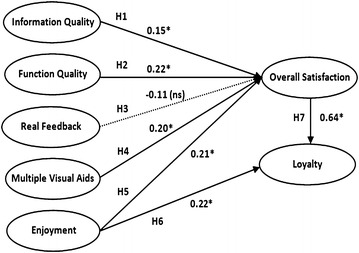
Tourism guide cloud service model and SEM results. *p < 0.05 and *ns* non-significant

As observed in Fig. 1 and Table 6, except for hypothesis H3 concerning the impact of real feedback on customers’ overall satisfaction, other six hypotheses were supported. Specifically, information quality (λ = 0.15*), function quality (λ = 0.22*), multiple visual aids (λ = 0.20*), and enjoyment (λ = 0.21*) were found to have positive effects on overall satisfaction. Additionally, enjoyment had positive impacts on overall satisfaction (λ = 0.21*) and overall satisfaction in turn exerted a positive influence on loyalty (λ = 0.64*). Furthermore, this result show that the standard error of path coefficient of real feedback on customers’ overall satisfaction is large (0.2). Hence, H3 was unsupported.

**Table 6:** Results of standard path coefficients for each hypothesis

Hypothesis	Standard path coefficient (SE)	Result
H1: information quality → overall satisfaction	0.15* (0.08)	Supported
H2: function quality → overall satisfaction	0.22* (0.09)	Supported
H3: real feedback → overall satisfaction	−0.11 (0.20)	Not supported
H4: multiple visual aids → overall satisfaction	0.20* (0.09)	Supported
H5: enjoyment → overall satisfaction	0.21* (0.09)	Supported
H6: enjoyment → loyalty	0.22* (0.05)	Supported
H7: overall satisfaction → loyalty	0.64* (0.04)	Supported

* p < 0.05

Furthermore, we also review the total effects of the factors on loyalty to gain a further understanding of the mediating effects of overall satisfaction. The results indicated that the most critical factor that affects loyalty of Tourism guide cloud service is enjoyment (total effect = 0.36, direct = 0.22, indirect = 0.14), followed by function quality (total effect = 0.14 = indirect), multiple visual aids (total effect = 0.13 = indirect), and information quality (total effect = 0.10 = indirect). Likewise, the effects of real feedback on loyalty are non-significant at *p* < 0.05. In other words, overall satisfaction effectively mediate Information quality, Function Quality, Multiple Visual Aids, and Enjoyment and significantly enhance loyalty of Tourism guide cloud service.

## Discussions and conclusions

In this customer-oriented era, to any service platform, the matters of how to improve, maintain, and manage the effective operation in order to keep current customers and capture potential users is the key issue for its sustainability. Overall, the results indicated that the most critical factor that affects loyalty of Tourism guide cloud service is enjoyment, followed by function quality, multiple visual aids, and information quality. On other hand, overall satisfaction effectively mediate Information quality, Function Quality, Multiple Visual Aids, and Enjoyment and significantly enhance loyalty of Tourism guide cloud service. These findings have beneficially suggested that service providers in designing and planning tourism guide cloud service platform should pay more attention to these aspects as well as manage them effectively once attempting to grasp customers’ satisfaction and use loyalty. In case the platform lacks of any of these aspects or there exist unsatisfactory issues, more investments for implementation and improvement are recommended.

With the achieved findings, this study has effectively extended the literature by investigating deeply the features of each factor, not just general understanding. As the most critical factor is enjoyment, users would be more excited and motivated in using a system once perceiving the system is fun and enjoyable (Chung and Tan 2004; Cyr et al. 2006; Moon and Kim 2001), the features of being “pleasant”, “attractive and full of curiosity”, and “interesting in content” have been well perceived to be critical to the success of tourism guide cloud services throughout the survey. Based on these premises, it is strongly suggested that guide cloud service providers should emphatically develop the playfulness and pleasure features during the stages of designing and planning the systems in order to better capture users’ positive attitude and interests.

Furthermore, function quality, multiple visual aids, and information quality are also important factors. In previous research concerning IT platforms, function quality has been examined through system’s storage capacity (Burda and Teuteberg 2014; Lee et al. 2014) and system functions such as effective usability, availability, reliability, and adaptability (Chen et al. 2015; DeLone and McLean 2004). This study is based on real demands of tourists in using guide cloud services has recognized that among the survey items, tourism guide cloud services’ capabilities in providing real-time information concerning traffic situations, points of interest and convenient stores as well as offering map navigation services have been considered important. Additionally, the abilities of instantly grasping messages related to sightseeing and helpful assistance as well as guiding references in trip planning are also the essential elements for customers in choosing tourism guide cloud services. For the fact that users tend to be dissatisfied once the platform cannot fulfill customers’ demands on these aspects, service providers are suggested to make more efforts in ensuring these features in order to maintain and enhance customer satisfaction and use intention.

Noteworthy, overall satisfaction is the mediated factor between Multiple Visual Aids and loyalty of Tourism guide cloud service. It is noted aforementioned that there remains the debate regarding the impacts of multiple visual aids on customer satisfaction in the extant literature between significant (Cyr et al. 2006; Lorenzo-Romero et al. 2013; Rosen and Purinton 2004; Tuch et al. 2010) and non-significant (Lee et al. 2014). Therefore, this finding has interestingly provided several academic contributions to the existing debate on the role of visual attraction-attributes and user interface design of cloud systems toward customer satisfaction. With this result, this study beneficially recommends cloud system providers and tourism managers to take multiple visual aids into deeper account once aiming to capture tourists’ satisfaction in using cloud services.

Surprisingly, real feedback is found to have non-significant influence on user satisfaction, which is contrast to previous studies that system’s fast-response feedback toward users’ complaints or requirements significantly promotes customer satisfaction (DeLone and McLean 2004) and that cloud system’s real-time travel assistances can increase customer satisfaction through presenting the latest information relevant to tourists’ needs and motivating their conversations with other users (Lee et al. 2014). In this study, it can be explained that since the shift from traditional IS services to cloud services is still in the early stage (Park and Ryoo 2013), it is hard for cloud tourism service providers to actually capture tourists’ feedback and expectation, thus its impact on customer satisfaction has yet been efficient.

As aforementioned, this study through innovatively integrating tourism industry and cloud services attempts to investigate which factors of cloud services (i.e. information quality, function quality, real feedback, multiple visual aids, and enjoyment) actually delight customer satisfaction and loyalty. In general, the results prove significantly positive effects of function quality, enjoyment, multiple visual aids, and information quality of cloud services on users’ overall satisfaction. Therefore, in order to increase tourists’ satisfaction and loyalty, it is essential for tourism guide cloud service providers and industrial managers to be satisfying brands and systems with which tourists can be able to feel favourably disposed. Managers are also recommended to be deeply concerned about tourists’ service expectations and feedback concerning these aspects for better motivating their satisfaction and loyalty.

As revealed, enjoyment plays the most critical role, followed by function quality and multiple visual aids, implying their crucial roles in determining customers’ satisfaction and loyalty, thus suggesting cloud system operators and industrial managers to strategically strengthen cloud platform’s conduct joyful tourism activities and function quality to maintain and enhance customers’ satisfaction and enjoyment toward the use of cloud services. As such, continuous use of cloud systems in tourism can be considerably ensured.

With the achieved findings, this study has beneficially provided several considerable academic and practical contributions to the extant literature with an extension of cloud service platforms in the field of tourism industry, which is originally a missing gap in previous research. As such, cloud services are not only understood in terms of their possible applications but also in the practical aspects needed by customers and the service gaps for improvement. Simultaneously, with the obtained real key factors, this study hopes to provide future research, academicians in relevant fields, and cloud service managers and providers with helpful references of research items of customer real needs and guidelines for enhancing tourism guide service quality in order to achieve better sustainable competitiveness.

### Research limitations and future recommendations

This study based on the Information Systems Success Model (DeLone and McLean 2004) and Media Richness Theory (Daft and Lengel 1986). In order to avoid complexity, this study only selected 7 constructs, including Information quality, Function Quality, Real Feedback, Multiple Visual Aids, Enjoyment, Overall Satisfaction, and Loyalty. In the future research, It is recommended to consider the other variables into the integrated model, e.g. technical quality, service quality, and so on. On the other hand, due to the development of Tourism Guide Cloud Service is still at the initial stage in Taiwan; this study focuses on the impact factors of Overall Satisfaction, and Loyalty of Tourism Guide Cloud Service. However, how to improve the Mobile Quality (including Service Quality, Information quality, Systems Quality) is also an important issue for the future development of Tourism Guide Cloud Service. It is recommended to study the improvement of Tourism Guide Cloud Service Quality for future research.

## References

[CR1] Amato F, Mazzeo A, Moscato V, Picariello A (2014) Exploiting cloud technologies and context information for recommending touristic paths, In: Intelligent distributed computing VII, Springer International Publishing, pp 281–287

[CR2] Bagozzi RP, Yi Y (1988). On the evaluation of structural equation models. J Acad Mark Sci.

[CR3] Bayraktar E, Tatoglu E, Turkyilmaz A, Delen D, Zaim S (2012). Measuring the efficiency of customer satisfaction and loyalty for mobile phone brands with DEA. Expert Syst Appl.

[CR4] Benlian A, Koufaris M, Hess T (2011). Service quality in software-as-a-service: developing the SaaS-qual measure and examining its role in usage continuance. J Manag Inf Syst.

[CR5] Bilgihan A, Okumus F, Nusair K, Kwun D (2011). Information technology applications and competitive advantage in hotel companies. J Hosp Tour Technol.

[CR6] Burda D, Teuteberg F (2014). The role of trust and risk perceptions in cloud archiving: results from an empirical study. J High Technol Manag Res.

[CR7] Chang HH, Chen SW (2008). The impact of customer interface quality, satisfaction and switching costs on e-loyalty: internet experience as a moderator. Comput Hum Behav.

[CR8] Chen LC, Tseng CY (2013). Managing service innovation with cloud technology. Global Bus Perspect.

[CR9] Chen JV, Yen DC, Pornpriphet W, Widjaja AE (2015). E-commerce web site loyalty: a cross cultural comparison. Inf Syst Front.

[CR10] Chou DC (2015). Cloud computing: a value creation model. Comput Stand Interfaces.

[CR11] Chung J, Tan FB (2004). Antecedents of perceived playfulness: an exploratory study on user acceptance of general information-searching websites. Inf Manag.

[CR12] Cyr D, Head M, Ivanov A (2006). Design aesthetics leading to m-loyalty in mobile commerce. Inf Manag.

[CR13] Daft RL, Lengel RH (1986). A Proposed integration among information requirements, media richness, and structural design. Manage Sci.

[CR14] Davis FD, Bagozzi RP, Warshaw PR (1989). User acceptance of computer technology: a comparison of two theoretical models. Manage Sci.

[CR15] DeLone WH, McLean ER (2004). Measuring e-commerce success: applying the DeLone & McLean information systems success model. Int J Electron Commer.

[CR16] Faed A, Hussain OK, Chang E (2014). A methodology to map customer complaints and measure customer satisfaction and loyalty. SOCA.

[CR17] Flavian C, Guinaliu M, Gurrea R (2006). The role played by perceived usability, satisfaction and consumer trust on website loyalty. Inf Manag.

[CR18] Fornell C, Larcker DF (1981). Structural equation models with unobservable variables and measurement error: algebra and statistics. J Mark Res.

[CR19] Gopalani A, Shick K (2011). The service-enabled customer experience: a jump-start to competitive advantage. J Bus Strat.

[CR20] Gudigantala N, Song J, Jones D (2011). User satisfaction with Web-based DSS: the role of cognitive antecedents. Int J Inf Manag.

[CR21] Hair JF, Black WC, Babin BJ, Anderson RE (2010). Multivariate data analysis.

[CR23] Hu L, Bentler PM (1998). Cutoff criteria for fit indexes in covariance structure analysis: conventional criteria versus new alternatives. Struct Equ Model.

[CR24] Kalloniatis C, Mouratidis H, Vassilis M, Islam S, Gritzalis S, Kavakli E (2014). Towards the design of secure and privacy-oriented information systems in the cloud: identifying the major concept. Comput Stand Interfaces.

[CR25] Kim HW, Kankanhalli A (2009). Investigating user resistance to information systems implementation: a status quo bias perspectivse. Manag Inf Syst Q.

[CR26] Lee SG, Yang CG, Lee SB, Lee JB (2014). A study on the antecedents and consequences of satisfaction and dissatisfaction in web portal usage. Serv Bus.

[CR27] Lin HH, Wang YS (2006). An examination of the determinants of customer loyalty in mobile commerce contexts. Inf Manag.

[CR28] Lin G, Fu D, Zhu J, Dasmalchi G (2009). Cloud computing: it as a service. IEEE IT Prof.

[CR29] Lorenzo-Romero C, Constantinides E, Alarcón-del-Amo MDC (2013). Web aesthetics effects on user decisions: impact of exposure length on website quality perceptions and buying intentions. J Internet Commer.

[CR30] Marston S, Li Z, Bandyopadhyay S, Zhang J, Ghalsasi A (2011). Cloud computing: the business perspective. Decis Support Syst.

[CR31] Martens B, Teuteberg F (2012). Decision-making in cloud computing environments: a cost and risk based approach. Inf Syst Front.

[CR32] Martin D, de Ipiña DL, Lamsfus C, Alzua A (2012) Involving tourism domain experts in the development of context-aware mobile services. E-rev Tour Res 10(3)

[CR33] Mohaupt M, Hilbert A (2013). Integration of information systems in cloud computing for establishing a long-term profitable customer portfolio. IAENG Int J Comput Sci.

[CR34] Moon J, Kim Y (2001). Extending the TAM for a world-wide-web context. Inf Manag.

[CR36] Nunnally JC (1978). Psychometric theory.

[CR37] Park SC, Ryoo SY (2013). An empirical investigation of end-users’ switching toward cloud computing: a two factor theory perspective. Comput Hum Behav.

[CR38] Petter S, DeLone W, McLean ER (2013). Information systems success: the quest for the independent variables. J Manag Inf Syst.

[CR39] Ratten V (2012). Entrepreneurial and ethical adoption behavior of cloud computing. J High Technol Manag Res.

[CR40] Raval V (2010). Risk landscape of cloud computing. ISACA J.

[CR41] Rosen DE, Purinton E (2004). Website design: viewing the web as a cognitive landscape. J Bus Res.

[CR42] Shin JI, Chung KH, Oh JS, Lee CW (2013). The effect of site quality on repurchase intention in Internet shopping through mediating variables: the case of university students in South Korea. Int J Inf Manag.

[CR43] Siddiqi KO (2011). Interrelations between service quality attributes, customer satisfaction and customer loyalty in the retail banking sector in Bangladesh. Int J Bus Manag.

[CR44] Song J, Baker J, Lee S, Wetherbe JC (2012). Examining online consumers’ behavior: a service-oriented view. Int J Inf Manag.

[CR45] Tseng SM (2015). Exploring the intention to continue using web-based self-service. J Retail Consum Serv.

[CR46] Tuch AN, Bargas-Avila JA, Opwis K (2010). Symmetry and aesthetics in website design: it’s a man’s business. Comput Hum Behav.

[CR47] Venkate sh V, Davis FD (2000). A theoretical extension of the technology acceptance model: Four longitudinal field studies. Manag Sci.

[CR48] Zhang L, Li N, Liu M (2012). On the basic concept of smarter tourism and its theoretical system. Tour Trib.

[CR49] Zhou Q, Hung JC, Hu J, Chen H, Zhou R, Qi J, Yang L, Wang X (2014) Cloud services aided e-tourism: in the case of low-cost airlines for backpacking. In: Frontier and innovation in future computing and communications. Springer, Netherlands pp 321–327

